# Peripheral arterial catheters in extremely preterm infants born at less than 28 weeks of gestation—a single-center experience

**DOI:** 10.1007/s00431-024-05699-w

**Published:** 2024-07-31

**Authors:** Marlies Bruckner, Michaela Schneider, Friedrich Reiterer, Lukas P. Mileder, Nariae Baik-Schneditz, Gerhard Pichler, Berndt Urlesberger, Bernhard Schwaberger

**Affiliations:** Division of Neonatology, Department of Pediatrics and Adolescent Medicine, Medical University of Graz, Graz, Austria

**Keywords:** Infant, Newborn, Preterm, Vascular access, Arterial catheter, Arterial monitoring

## Abstract

The aim of this study was to perform a retrospective data analysis of established peripheral artery catheters (pAC) in extremely preterm infants. The primary outcome was the pAC life span and its correlation to gestational age, birth weight, localizations, and pAC removal. Retrospective data analysis of electronic patient records of all extremely preterm infants (born less than 28 weeks gestation) admitted to the neonatal intensive care unit in Graz (Austria) between January 2014 and December 2020. A total of 196 preterm infants with a median (IQR) gestational age of 25.7 (24.6–26.6) weeks and a birth weight of 730 (614–898) g were included. In 155 (79%) of these preterm infants, 286 pAC and six umbilical artery catheters were inserted successfully. The first pAC was inserted 2.5 (1.4–7.4) h after birth, and the median pAC life span was 57.5 (22.–107.2) h. Gestational age, birth weight, and catheter localization did not correlate with the pAC life span. The pAC localizations were the radial artery (63%), tibial posterior artery (21%), ulnar artery (6%), dorsal artery of the foot (6%), others (1%), and not documented (3%). Adverse reactions including temporarily impaired peripheral perfusion, local inflammation, extravasation, or bleeding were reported in 13% of all pAC, but none of these resulted in long-term sequelae. A median (IQR) of 9 (5–18) arterial blood samples were drawn via pAC, resulting in a notable reduction of pain stimuli.

*Conclusion*: The use of pAC in extremely preterm infants is feasible and safe. Neither gestational age, birth weight nor localization did affect the life span of pAC. No long-term sequelae were observed, and pain events were reduced by using pAC for blood drawing.

**Table:** 

**What is Known:** • *Peripheral artery catheters can be used for continuous blood pressure measurement and blood draw even in extremely preterm infants*. • *(Severe) adverse reactions such as bleeding, necrosis, or amputation occur between 1 and 4%*. • **What is New:** • *The median peripheral arty catheter life span is 58 h and is not affected by gestational age, birth weight, nor localization*. • *A median of nine blood samples can be taken per each single pAC and, therefore, prevent pain events in extremely preterm infants*.

**What is Known:**

• *Peripheral artery catheters can be used for continuous blood pressure measurement and blood draw even in extremely preterm infants*.

• *(Severe) adverse reactions such as bleeding, necrosis, or amputation occur between 1 and 4%*.

• **What is New:**

• *The median peripheral arty catheter life span is 58 h and is not affected by gestational age, birth weight, nor localization*.

• *A median of nine blood samples can be taken per each single pAC and, therefore, prevent pain events in extremely preterm infants*.

## Introduction

Peripheral artery catheters (pAC) are an established alternative for arterial vascular access in newborn infants. pAC are used for continuous invasive blood pressure measurement and blood gas monitoring in neonatal intensive care patients. Once a pAC has been established, it enables arterial blood drawing and repetitive blood gas analysis without causing additional pain. Nonetheless, due to the possible serious complications such as embolization, ischemia of the extremities, or bleeding, there should be a strict indication for pAC establishment [1]. However, the use of a pAC is cost-effective and rather simple, and they can be used even in extremely preterm infants [2]. Preferred locations described in the literature for pAC in newborn infants are the radial artery (ideally on the right arm to be able to take blood preductally), the posterior tibial artery, the ulnar artery, the brachial artery, the dorsal artery of the foot, and, in some cases, the femoral artery [1, 3–5]. According to localization and gestational age, severe complication rates vary between 1 and 4% [1, 6, 7].

Although frequently used, only little is known about the pAC life span, favorable localizations, reasons for catheter removal, adverse event rates, and number of arterial blood draws per pAC, particularly in extremely preterm infants. The aim of this study was to perform a retrospective data analysis of all established pAC in extremely preterm infants admitted to the neonatal intensive care unit (NICU) in Graz, Austria, within a period of seven years. The primary outcome was the pAC life span and its correlation to gestational age, birth weight, localizations, and pAC removal.

## Methods

A retrospective data analysis was conducted at the Division of Neonatology, Medical University of Graz, Austria, including preterm infants born at less than 28 weeks of gestation who were admitted to the NICU between January 2014 and December 2020. The study was approved by the ethics committee of the Medical University of Graz (ethics committee number: 32–135 ex 19/20) and conducted in accordance with the Declaration of Helsinki.

### Data collection

Demographic data including gestational age, birth weight, sex, umbilical artery pH, Apgar scores, and short-term outcome (including death) were extracted from the local database. A retrospective medical chart review was accomplished using openMEDOCS (Steiermaerkische Krankenanstaltengesellschaft, Graz, Austria) and Patient Data Management Systems Centricity™ Critical Care 9.0 SP1 (General Electric Company, Boston, MA, USA). Extracted data included the number of pAC per preterm infant, postnatal age at insertion, pAC life span (time period from inserting the catheter until removal), localization, reasons for removal (loss of function, ischemia, weeping puncture site, paravasation, accidental removal, local inflammation, no further need if the preterm infants’ condition improved or the infant died, and unclear reasons), complications and adverse reaction, and number of blood draws per pAC. Data were extracted and collected in an Excel spreadsheet (Microsoft, Redmond, WA, USA).

### Peripheral artery catheter insertion

**Fig. 1:**
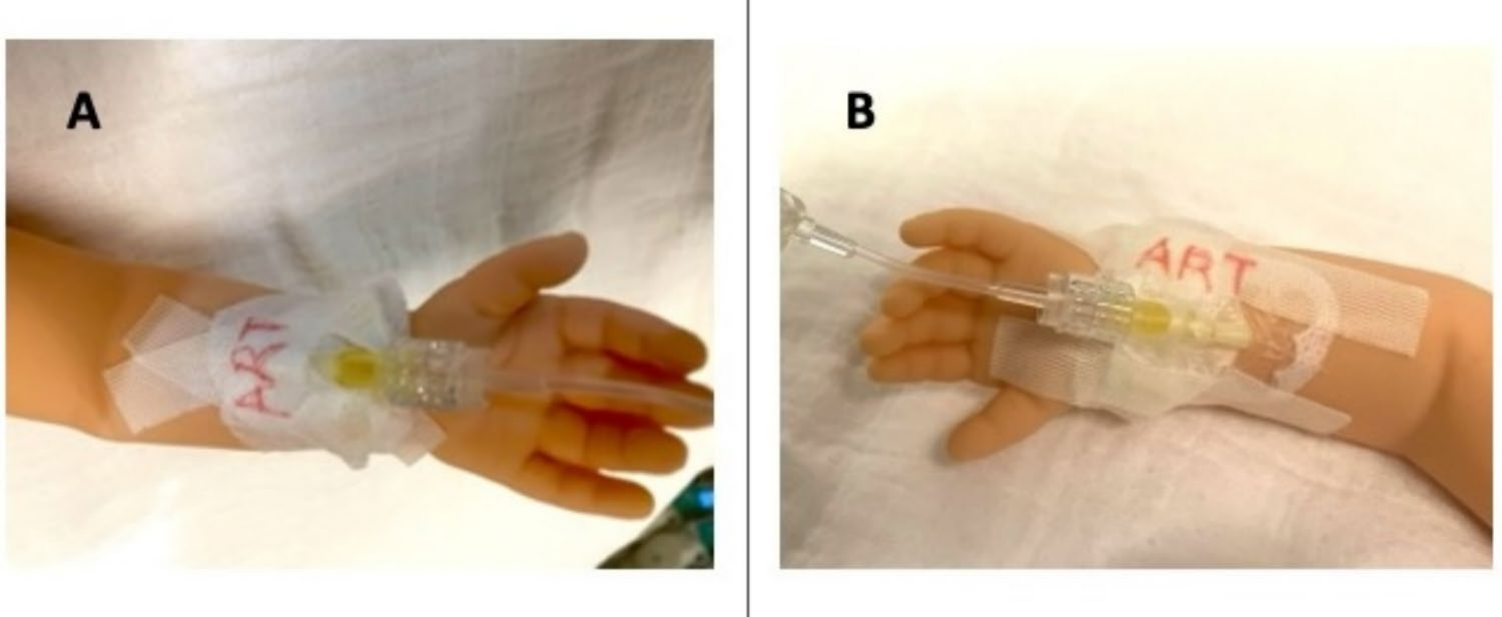
**A** Catheter fixation technique with sterile stripes. **B** Catheter fixation technique with a transparent semipermeable membranes

pAC insertion is a standardized procedure at our NICU. It is always performed under sterile conditions either by a resident under supervision or by a neonatologist. The procedure is usually performed in the incubator, and we use sterile cloths, sterile gloves, a sterile field, and only sterile equipment to keep the environment as aseptic as possible. For skin disinfection, 0.1% octenidin-dihydrochlorid without phenoxyethanol is used [8, 9]. The preferred localization is the radial artery. Before radial artery puncture, Allen’s test is performed in all infants to test for the presence of a collateral circulation in the hand and to avoid impaired peripheral perfusion [10]. Infants receive non-nutritive sucking, glucose, and/or facilitated tucking for pain management during pAC insertion. After hand fixation, the skin at the puncture site is stretched, and the puncture of the artery is performed with a Becton Dickinson Neoflon™ (Franklin Lakes, NJ, USA) catheter—the size of 24 or 26 G—in an angle of 10 to 15 degrees to the skin surface. A strong red-light source (Wee SightTM, Philips, The Netherlands) is used for transillumination to visualize pulsation and the course of the peripheral artery [11]. After correct catheter placement, the mandrin is removed, and the catheter is flushed with not more than 0.5–1 ml of normal saline. The catheter is fixed with sterile dressing material (Fig. 1), and an infusion with normal saline and double-distilled water (1:1) and heparin (1 I.E./ml) is started with a flow rate of 0.5–1.0 ml/h.

### Statistical analysis

Data are presented as mean (SD) for normally distributed continuous variables and median (IQR) when the distribution was skewed. Data were tested for normality (Shapiro–Wilk and Kolmogorov–Smirnov test). Correlation coefficients were generated using Pearson and Spearman correlation analyses. A *p*-value < 0.05 was considered statistically significant. The analyses were performed using Excel software (Microsoft, Redmond, WA, USA) and IBM SPSS Statistics 26 (IBM, Armonk, NY, USA).

## Results

One hundred ninety-six preterm infants born at less than 28 weeks of gestation were admitted to the NICU, Graz, between January 2014 and December 2020. One hundred fifty-two (78%) of those received a total number of 286 pAC during hospitalization. In six preterm infants, umbilical artery catheters (UAC) were inserted successfully after birth, and three of them additionally received a pAC in the further clinical course. Demographic data and pAC characteristics are displayed in Table 1.

**Table 1:** Demographics and peripheral arterial catheter characteristics. *pAC*, peripheral artery catheter; *h*, hours. Data are expressed as median (IQR), mean (SD), or *n* (%)

	Total
Demographics	
*n*	196
Gestational age (weeks)	25.7 (24.6–26.6)
Birth weight (g)	730 (614–898)
Female sex *n* (%)	92 (47)
Small for gestational age *n* (%)	27 (14)
Mortality *n* (%)	38 (19)
Death on first day after birth *n* (%)	9 (24)
Late onset sepsis *n* (%)	3 (2)
Cord arterial pH	7.29 (7.24–7.36)
Apgar 5	8 (7–9)
Characteristics of pAC	
Infants with pAC *n* (%)	152 (78)
Number of inserted pAC	286
Number of pAC per infant	1.5 (1.4)
Age at first insertion (h)	2.5 (1.4–7.4)
pAC life span (h)	58 (22–107)

### Characteristics of peripheral artery catheters

Considering all preterm infants admitted to the NICU during the study period (*n* = 196), a median (IQR) of 1(1–2) pAC was inserted per infant. The maximum number of pAC inserted during the NICU stay in a single patient was eight. The first pAC was inserted at a median (IQR) of 2.5 (1.4–7.4) h after birth and the median pAC life span was 57.5 (22.5–107.2) h. The maximum life span of a single pAC was 440 h. There was no correlation between the pAC life span neither with gestational age (*ρ* = 0.061; *p* = 0.461) nor with birth weight (*ρ* =  − 0.061; *p* = 0.487). However, infants with a lower gestational age received a higher number of pAC and pAC at an earlier time compared to infants with a higher gestational age (Table 1). pAC were most frequently inserted into the radial artery (63%), followed by the posterior tibial artery (21%), the dorsal artery of the foot (6%), the ulnar artery (6%), the brachial artery (0.4%), and the femoral artery (0.4%) (Table 2). There was no correlation between pAC localization and pAC life span (*ρ* = 0.123; *p* = 0.643).

**Table 2:** Peripheral artery catheter localization and life span depending on localization. Data are expressed as median (IQR) or *n* (%)

	*n* (%)	Life span (h)
*Peripheral artery catheter localization*		
Radial artery	181 (63)	54 (22–109)
Posterior tibial artery	61 (21)	47 (27–113)
Dorsal artery of the foot	17 (6)	53 (8–95)
Ulnar artery	16 (6)	24 (15–89)
Brachial artery	1 (0.4)	3
Femoral artery	1 (0.4)	54
Missing data of chosen catheter localization	9 (3)	46 (23–67)
Total	286 (100)	51 (21–105)

A median (IQR) of 9 (5–18) blood draws were conducted per single pAC. The maximum number of blood draws through a single pAC (with a life span of 317 h) was 67. In total, 3741 blood draws through pAC were documented in the 152 preterm infants. Hence, approximately 25 blood samples were taken per infant through the pAC, avoiding a relevant number of painful events.

### Adverse reactions and reasons for peripheral artery catheter removal

A total of 35 (13%) adverse reactions were observed including local signs of malperfusion (e.g., paleness of the skin) (*n* = 14, 40%), local inflammation (*n* = 11, 31%), paravasation (*n* = 6, 17%), and bleeding at the puncture site (*n* = 4, 12%). No serious adverse reaction, such as fingertip necrosis, occurred. All complications regressed, and no long-term sequelae were observed.

Reasons for removal of pAC were loss of function (*n* = 118, 41%), ischemia (*n* = 48, 17%), weeping puncture site (*n* = 10, 4%), paravasation (*n* = 6, 2%), accidental removal (*n* = 4, 1%), no further need (*n* = 9, 3%), local inflammation (*n* = 2, 1%), and not documented or unclear (*n* = 89, 31%). There was no correlation between reasons for removal and pAC life span (*ρ* = 0.185; *p* = 0.386).

## Discussion

pAC can be used beside UAC for arterial catheter management in newborn infants. However, there is a lack of data about the use and safety of pAC, especially in extremely preterm infants. Therefore, we reviewed in the present study all pAC in extremely preterm infants born at less than 28 weeks of gestation admitted to the NICU, Graz, over a 7-year period. Our results can be summarized as follows: (i) pAC insertion within the first 3 h after birth is feasible and safe in extremely preterm infants; (ii) pAC remained in situ for a median of 58 h; (iii) there was no association between the pAC life span and either gestational age, birth weight, localization, or reason for removal; (iv) the preferred localization was the radial artery, followed by the posterior tibial artery; (v) pAC were used for multiple blood draws and, therefore, reduced painful events; (vi) all observed adverse reactions were temporary and did not lead to long-term sequelae.

### Peripheral artery catheter insertion and lifespan

In the present study, the pAC life span was a median of 58 h. There are limited data to compare our results to the literature since most publications report the total pAC insertion time. Deindl et al. reported a median total time with indwelling pAC between 2 and 3 days within the first week after birth and 3 days after the first week after birth [1]. However, they reported the total time of indwelling pAC without detailing how long a single catheter remained in situ [1]. It was described that in preterm infants below 1000 g, the mean (range) duration of indwelling pAC was 86 (24–240) h in electively removed pAC and mean (range) 101 (1–231) h in early removed catheters [5]. These time periods using the same catheter size are longer than in our study, which may be explained by their higher heparin dose and flow rate (1–2 I.E./ml at a rate of 2 ml/h vs. 1 I.E./ml at a rate of 0.5–1 ml/h). However, the number of infants below 1000 g in the study by Randel et al. was small *(n* = 35), and it is not clearly stated whether the time period was calculated for each preterm infant or for each pAC [5].

Imamura et al. compared pAC versus UAC insertion in 38 very preterm infants [12]. They reported that the first pAC was inserted 3.0 (1.5–10.5) h after birth which did not differ from UAC insertion and is in concordance with our data [12]. Although they stated that the median times of catheter indwelling were similar between the groups, unfortunately, they did not report specific data about the time period from pAC insertion to removal [12]. Very recently, Mense et al. analyzed data from pAC and UAC insertion in preterm infants born at less than 26 weeks of gestation [7]. Because their NICU policy changed during the last 10 years (primary arterial access changed from UAC to pAC), two cohorts were compared [7]. In those preterm infants in whom pAC was the primary arterial access, its life span was a median of 4 days, and it was inserted at 0.40 (0.1–2.39) days after birth [7]. This was considerably longer than in the present study, although the same fluid management was used. However, there are no international recommendations or guidelines available for arterial lines (including fluid management) in preterm infants. In addition, also the use and dosage of heparin, the aseptic technique, equipment and catheter size, insertion technique, choice of blood vessel, catheter fixation, arm or leg immobilization, nursing procedures, and health care providers’ experience are factors that influence the arterial line life span [13–16].

The present analyzed data showed that the lower the gestational age, the earlier the pAC was inserted. This was probably due to the increased need for respiratory support or higher incidences of cardiocirculatory instability, as shown by the higher number of patients requiring catecholamines, in the more immature preterm infants. However, there was no association between pAC life span and gestational age or birth weight. This was also observed in another study [5]. This result was surprising, as we expected the smaller diameters of various arterial vessels at lower birth weights and lower gestational ages to influence the time from pAC insertion to removal. It is, therefore, possible that it is not the low gestational age and birth weight but rather the selected vessel that is the decisive factor for the occurrence of adverse reactions [6, 15, 17].

### Peripheral artery catheter localizations

In this study, the preferred localization was the radial artery, followed by the posterior tibial artery. There are some studies describing the brachial artery as the second puncture site after the radial artery [3, 4]; however, there are data that the posterior tibial artery is a good alternative as a second choice [1, 5]. The brachial artery is the only major artery on the upper arm, and in the event of an injury or thrombotic vessel occlusion, severe ischemic complications might result even in whole-arm amputation [4]. In 615 children at a median age of 0.56 years, it was demonstrated that thrombosis is not only associated with lower age but also with pAC localization [6]. Thrombosis occurred more often at the femoral localization compared to the radial artery, which should be preferred over the femoral artery [6, 15, 17]. Besides these considerations, health care providers’ experience and local standards should also be considered when choosing the insertion site.

The median pAC life span at the radial artery was the longest, although this was not statistically significant. Only 1% of locations were unusual; hence, this would have not changed results excluding them. However, there was no association between the pAC localization and life span. To the best of our knowledge, there are no other studies reporting on the association of catheter life span and puncture site published until now.

### Reduced pain events

Exposure to pain in the neonatal period might result in adverse neurodevelopmental outcomes [14, 18, 19]. The newborn infants included in the present study received their first pAC within the first 3 h after birth. Especially during the first days after birth, frequent blood gas analyses are needed due to respiratory and metabolic instability. Previously, it has been reported that the use of pAC for the routine treatment of high-risk neonates in NICUs is an effective way to reduce the level of suffering, by decreasing the need for venipuncture and capillary blood collection from the heel [12]. Our analysis showed that a median of nine blood samples could be taken per single pAC. Considering the whole hospital stay, 25 blood samples were drawn per infant through pAC. This result is particularly noteworthy, because it is the first such evaluation of prevented pain events in extremely preterm infants in the literature. So far, there are no comparative values from other studies, and our data can serve as a reference point for comparing blood sampling procedures in future work on pain avoidance in preterm neonates.

### Adverse reactions and reason for removal

Generally, newborn infants with a lower birth weight and a lower postmenstrual age have an increased risk of adverse events from peripheral arterial catheters [1]. Acral ischemia is a serious complication and might lead to tissue necrosis, which can ultimately result in (partial) amputation of the extremities [1].

The present data show that the use of pAC even in extremely preterm infants is feasible and safe. It is noteworthy that there were no long-term sequelae observed in association with the placement of pAC during the time period of 7 years. Deindl et al. reported on severe adverse reactions of 542 pAC in 485 preterm infants (thereof, 275 born at less than 28 weeks of gestation) with a complication rate of 4%, reporting only on severe ischemia, severe tissue damage, or required surgery [1]. Indeed, in 44% of those preterm infants with complications, deep tissue necrosis resulting in amputation had occurred, and three (19%) patients needed surgical amputation, necrosectomy, and split-thickness skin grafting [1]. Other studies also reported on severe complication rates between 1 and 4% including thrombosis and severe ischemia [6, 7]. This contrasts with the present study, where no serious adverse events were reported. To the best of our knowledge, there is only one other study with no long-term complications; however, the number of included extremely preterm infants was very low, and they did not include infants with a birth weight below 750 g [2].

There is very limited data about the reasons for removal. While in the present study, only 3% of pAC were removed because they were no longer needed, and other studies reported elective removal in between 17 and 42% [5, 7]. The main reasons for early removal in the present study were loss of function followed by ischemia, which is in concordance with the literature [5, 7]. In our study, the pAC life span was shorter, the rate for early removal was higher, and there was no occurrence of serious adverse reactions compared to the literature. We can only speculate that the local health care providers’ awareness and standards may be the reasons behind the shorter life span and earlier removal. This approach could have contributed to the low serious adverse reaction rate without any long-term damage compared to other studies.

### Strengths and limitations

The strength of this study is the large number of extremely preterm infants, with a total of 286 included pAC representing a relevant sample size. In addition, this is the first study reporting on reduced pain events due to the insertion of pAC which has an impact on further investigations.

One limitation is the retrospective study design. However, the partial lack of data on localization and the reason for removal are assumed to not change the present results and their clinical relevance substantially. Another limitation is that there was no data about the attempts to insert a pAC, which must be considered when interpreting the results. However, since pAC insertion is a standard procedure in our NICU and health care providers are well trained in this intervention, we assume that our success rate with the first attempt is similar to 82% [2].

## Conclusion

The use of pAC in extremely preterm infants is feasible and safe, with a median pAC life span of 58 h. There was no association between gestational age, birth weight, localization, reason for removal, and the life span of pAC. No long-term sequelae occurred in association with the indwelling pAC. This is the first study reporting on the numbers of up to 25 reduced pain events per infant by averting heel punctures or venous punctures using pAC for blood draw.

## Data Availability

The data that support the findings of this study are available through the corresponding author.

## Abbreviations

h: Hour(-s)
IQR: Interquartile range
NICU: Neonatal intensive care unit
pAC: Peripheral artery catheter(-s)
SD: Standard deviation
UAC: Umbilical artery catheter(-s)
